# Waist to Height Ratio and Metabolic Syndrome as lung dysfunction predictors

**DOI:** 10.1038/s41598-020-64130-0

**Published:** 2020-04-29

**Authors:** Rafael Molina-Luque, Manuel Romero-Saldaña, Carlos Álvarez-Fernández, Enrique Rodríguez-Guerrero, Alberto Hernández-Reyes, Guillermo Molina-Recio

**Affiliations:** 1 Department of Nursing, Faculty of Medicine and Nursing, University of Córdoba, Av. Menéndez Pidal No/No, 14004 Córdoba, Spain; 2 Department of Occupational Safety and Health, Córdoba City Hall, Huerto San Pedro el Real Street, 1, 14003 Córdoba, Spain; 3 UGC Lucena, Andalucía Health Service, Calle Paseo de Rojas, 0, 14900 Córdoba, Spain

**Keywords:** Cardiovascular diseases, Metabolic disorders, Respiratory tract diseases, Risk factors, Health care

## Abstract

Metabolic Syndrome (MetS) has been related to pulmonary diseases but its relationship with lung age has not been sufficiently studied. In addition, anthropometric variables have been associated with pulmonary dysfunction, highlighting the waist-to-height ratio (WHtR). The aim was to evaluate the relationship between MetS and: lung age, anthropometric variables and the alteration of lung function. A cross-sectional study was carried out in 1901 workers, evaluating lung function through lung age (Morris & Temple equation) and spirometric values. The diagnosis of MetS was based on the harmonized criteria. We measured anthropometric variables (WHtR, waist circumference, body mass index, waist to hip ratio), blood pressure and biochemical variables (glucose, cholesterol total, HDL, triglycerides). Workers suffering from MetS showed an accelerated lung aging (59.4 ± 18.7 years vs 49 ± 18.4 years). The WHtR ≥ 0.55 was significantly related to an increase in lung age (β = 6.393, p < 0.001). In addition, a significant linear trend was found between clinical categories of WHtR and lung dysfunction, restrictive and mixed pattern. MetS caused an accelerated lung aging and favored the presence of restrictive lung impairment. In addition, WHtR ≥ 0.55 has been shown as the best predictor for pulmonary health.

## Introduction

Metabolic Syndrome (MetS) is defined as a group of cardiometabolic disorders that increase the risk of suffering from cardiovascular diseases and type II diabetes mellitus. Its prevalence is over 30% in several countries^1–3^. Abdominal obesity and insulin resistance are a key factor in the development of the characteristic alterations of MetS. Highlights include increased blood pressure, mixed dyslipidemia and impairment of different organs^4,5^. Several studies have shown in recent years that insulin resistance and MetS could be associated with alterations in lung function. This has been observed both in subjects with established respiratory pathologies and in healthy populations, and it is associated with the development of asthma, restrictive diseases or chronic obstructive pulmonary disease^6,7^. Pulmonary dysfunction is related to the appearance of cardiovascular diseases, both of them being among the leading causes of death worldwide. This has a high impact on the quality of life of people and the economic burden on health systems^8–11^.

Therefore, early diagnosis of MetS and lung dysfunction is necessary for the prevention of cardiovascular complications and mortality associated with both diseases. However, the invasiveness or diagnostic complexity of the methods used complicates their application in the clinical setting. Concerning MetS, diverse works have developed instruments that facilitate its diagnosis through the use of anthropometric measurements. The waist-to-height ratio (WHtR) has been postulated as the best predictive measure for MetS for a cut-off point of 0.55^12,13^.

Spirometry is the test of election for the assessment of lung function. For the interpretation of the results it provides, reference formulas for the calculation of normal or standard values are required. However, there is a wide variety of estimation equations, such as the *Global Lung Initiative*, which means that clinicians have to choose one among several. Also, they must take into consideration that not all are validated for all populations^14,15^. All this could make it difficult for health care professionals to make a correct assessment^16^.

In this sense, lung age, a parameter proposed for motivation in smoking cessation, is an easily interpreted variable by health professionals and patients for the early detection of pulmonary dysfunction. Several studies have demonstrated that lung age is associated with the severity of asthmatic pathology and that it is sensitive to systemic pathological processes such as overweight and obesity, causing the aging of the lungs. However, their possible relationship with MetS has not been established at present^17–19^.

As with MetS, the literature has shown how anthropometric indices of central obesity are associated with respiratory dysfunction. Nevertheless, despite the strong association that the waist-to-height ratio (WHtR) has shown with the alteration of lung function in healthy adults and the pediatric population, the cut-off point of 0.55 has not been assessed^20–22^.

Therefore, the main objective of the study is to determine whether MetS is associated with respiratory dysfunction as measured by lung age. The initial hypothesis is that those subjects suffering from MetS present a higher lung aging. Also, the aim is to assess what type of pulmonary dysfunction is most prevalent amongst sufferers of MetS and to establish which of its components, anthropometric variables and pathological situations are significantly associated with lung aging.

## Material and Methods

### Study design and sample

We carry out a cross-sectional study on the working population of Córdoba City Council (Spain) that employs an average of 1,760 workers per year. The minimum sample size calculated was of 1,685 workers for a power of 80%, a precision of 1.5%, a confidence of 95%, and an expected prevalence of MetS of 14.9%. The final sample, stratified by age and sex, was composed of 1,901 workers selected randomly among those who had undergone an occupational health examination at the City Council Occupational Health Unit between 2015 and 2019.

The exclusion criteria were to suffer from an acute or chronic pulmonary pathology (asthma, chronic bronchitis, emphysema, pneumonia, chronic obstructive pulmonary disease) or any other pathology affecting lung function; and not to be capable of performing the spirometric test adequately. The total number of workers excluded due to pulmonary pathology was 168. Moreover, according to the specific occupational risk assessment of the workplaces, none of the selected workers was exposed to working conditions (risk of inhalation of inorganic or organic dust, aerosols, or substances of high molecular weight), which could cause the appearance of occupational pulmonary pathology.

### Study variables

Pulmonary function (result variable) was studied through the lung age calculated by the formula proposed by Morris *et al*.^17^.

$$\begin{array}{c}{\rm{Women}}:{\rm{Lung}}\,{\rm{age}}({\rm{years}})=4.792\times {\rm{height}}-(41.667\times {\rm{FVC}})-118.883\\ {\rm{Men}}:{\rm{Lung}}\,{\rm{age}}({\rm{years}})=5.920\times {\rm{height}}-(40.00\times {\rm{FVC}})-169.640\end{array}$$

The height is expressed in inches (1 inch = 2.54 cm) and the forced vital capacity (FVC) in liters. We established the minimum lung age at 20 years, with no limitation on the maximum one. The difference between the lung age and the chronological age of the workers was calculated^18^.

Also, the spirometric parameters were collected: forced expiratory volume in the first second (FEV_1_), FVC and their quotient (FEV_1_/FVC). Theoretical values were calculated for each of the parameters in the aim of finding out the relationship with the values obtained in practice (FEV_1_% of predicted and FVC% of predicted). The patients’ lung function status was classified according to the following criteria^23^: Normality: FEV_1_/FVC > 0.7 and %FVC of predicted> 0.8; restrictive pattern: FEV_1_/FVC > 0.7 and %FVC of predicted <0.8; Obstructive alteration: FEV_1_/FVC < 0.7 and %FVC of predicted> 0.8; Mixed pattern: FEV_1_/FVC < 0.7 and %FVC of predicted <0.8.

The independent variables collected were age, gender and those grouped into:

- Lifestyles: smoking habit (non-smoker, ex-smoker and smoker); and physical activity (International Physical Activity Questionnaire, IPAQ) measured in METs and categorized as light, moderate and intense.
- Anthropometric variables: weight (kg), height (cm), body mass index (kg/m^2^, BMI), waist circumference (cm, WC), hip circumference (cm, HC), body fat percentage (Deurenberg), waist to hip ratio (WHR), waist to height ratio (WHtR), systolic and diastolic blood pressure (SBP and DBP, mmHg). Also, we considered three clinical categories for the WHtR (≤0.5; 0.51–0.54, ≥0.55) following the cut-off points proposed by Romero-Saldaña *et al*.^12,13^ and Ashwell *et al*.^24^. For the categorization of the BMI, the recommendation established by the World Health Organization was followed.
- Analytical variables: fasting plasma glucose (mg/dl), HDL-cholesterol (mg/dl) and triglycerides (mg/dl).

### Evaluation and measurement of variables

MetS diagnosis was established based on the harmonized criteria that define it by the presence of three or more of the following alterations. WC ≥ 102 cm in men, WC ≥ 88 cm in women; High Blood Pressure (HBP) ≥ 130/85 mmHg or undergoing treatment for high blood pressure; triglycerides ≥ 150 mg/dl; HDL < 40 in women, and <50 in men or being under pharmacological treatment; and fasting glucose ≥ 100 mg/dl or undergoing hypoglycemic treatment or diagnosis of Diabetes Mellitus^1^.

We obtained the spirometric values using the spirometer DATOSPIR 120 C (Silbemed; Barcelona; España), following the recommendations established by the Spanish Society of Pneumology and Thoracic Surgery (SEPAR)^23^. For the measurement, the worker was required not to use bronchodilators, not to smoke and not to do physical exercise in the previous hours. The test was performed with the worker sitting down, with his back straight and leaning against the chair back, and with a nose clip attached. We asked to inhale as much air as possible and to blow fast and strongly until it was indicated. The workers performed at least three acceptable tests, and the highest spirometric values were selected. We used the criteria of SEPAR to consider spirometry acceptable: rapid start (back-extrapolated volume less than 5% of the FVC) and without hesitation; continuous expiratory test with a duration not less than 6 seconds; no abrupt end (final changes in volume lower than 0.025 l for ≥1 s; and no anomalies in the technique (cough, new inhalation, among other). Two or three acceptable maneuvers were necessary for its interpretation in which the difference between the two best FVC and FEV1 was not more than 0.2 l.

The anthropometric measurements were recorded following the recommendations in the standardized anthropometry reference manual^25^. The weight and height were measured using a stadiometer and Atlántida S11 balance (Básculas y Balanzas Añó-Sayol, Barcelona, Spain), with a precision of 0.1 kg and 0.1 cm, respectively. WC was measured halfway between the last rib and the iliac crest after a normal exhalation. HC was measured at the widest point on the buttocks. Both measurements were taken with the worker standing, feet together, using a flexible steel tape. Blood pressure was measured as recommended in the Manual of Hypertension in Primary Care Clinical Practice^26^, using a calibrated digital sphygmomanometer (OMRON M3, OMRON Healthcare Europe, Spain). All the measures were recorded by experienced staff to decrease the variation coefficient, repeating each one three times and calculating the mean^12,13^.

### Legal and ethical aspects

All the workers were informed, verbally and in writing, of the objectives of the health study to which they were being submitted, and an informed consent was obtained from each in compliance with the current regulations. The study’s protocol complied with the Declaration of Helsinki for conducting research involving human subjects and was approved by the Bioethical Committee of Córdoba (Spain) (4427/Acta number 295).

### Statistical analyses

The quantitative variables are presented as the mean and standard deviation, the qualitative values presented as frequencies and percentages^27^.

To contrast the goodness-of-fit to a normal distribution of the data from the quantitative variables, the Kolmogorov–Smirnov test with the Lilliefors correction was employed. The Student’s t-test for two means was performed for the bivariate hypothesis contrast, whereas for the qualitative variables, the z-test for independent proportions and the Chi-square and Fisher exact test were used when necessary. In addition, for the correlation between the quantitative variables, the Pearson’s correlation coefficient (r) was used^27^. The analysis of covariance (ANCOVA) was made to diminish the heterogeneity produced by the influence of the chronological age on lung age.

Adjusted multiple linear regressions were made according to age, gender, smoking habits, and physical activity, with each explanatory variable to determine their prediction capacity for lung age. To determine the goodness-of-fit of the models, we analyzed the standard error, the adjusted coefficient of determination, the F statistic, the linearity analysis, and the residues.

For all the statistical analyses, the probability of an α error of below 5% (p < 0.05) was considered statistically significant and the confidence interval was calculated at 95%. For the statistical analysis, IBM SPSS Statistics 22.0 software (IBM, Chicago, IL, USA) and Epidat 4.2. (Department of Sanidade, Xunta de Galicia, Galicia, Spain) were used.

## Results

### Sample description

Out of a total of 1,901 workers, 51.3% (95% CI 49–53.6%) were women. The mean age was 43.6 ± 10.9 years (95% CI 43.1–44.5 years). The mean BMI was 26.7 ± 5.4 kg/m^2^ (95% CI 26.5–27 kg/m^2^). Based on the criteria established by the BMI, the prevalence of overweight (BMI ≥ 25 kg/m^2^) rose to 35% (95% CI 32.8–37.2%) and of obesity (BMI ≥ 30 kg/m^2^) to 22.9% (95% CI 21–24.8%).

The proportion of workers who smoked was 34.3% (95% CI 32.2–36.5%), being higher in women than in men (p < 0.001). The prevalence of HBP, type 2 diabetes mellitus and MetS reached 38.9% (95% CI 36.7–41.2%), 6% (95% CI 5–7.2%) and 18.3% (95% CI 16.5–20.1%), respectively. In all cases, it was significantly higher in men (p < 0.05).

Regarding pulmonary function status, the restrictive lung impairment was the most prevalent (13.5% [95% CI 12–15.1%]), with differences observed by sex (p < 0.01). Finally, lung age was 50.8 ± 20.5 years (95% CI 49.9–51.7 years), being higher in men (p < 0.001).

Table 1 contains a summary of all the variables collected stratified by sex.

**Table 1:** Sample characteristics.

Variables	Total (n = 1901)	Women (n = 975–51.3%)	Men (n = 926–48.7%)	p
Age (n = 1901)	43.6 (SD 10.9)	41.6 (SD 10.5)	45.6 (SD 10.9)	<0.001
Height (n = 1901)	167.8 (SD 9.4)	161.3 (SD 6.3)	174.8 (SD 6.7)	<0.001
Weight (n = 1901)	75.6 (SD 17.3)	68.4 (SD 15.8)	83.1 (SD 15.5)	<0.001
BMI (n = 1901)	26.7 (SD 5.4)	26.3 (SD 6)	27.2 (SD 4.7)	<0.01
Underweight	42 (2.2%)	33 (3.4%)	9 (1%)	<0.001
Normo-weight	759 (39.9%)	452 (46.3%)	307 (33.1%)	
Overweight	665 (35%)	263 (27%)	402 (43.4%)	
Obesity	435 (22.9%)	227 (23.3%)	208 (22.5%)	
Waist (n = 1844)	89.5 (SD 14.2)	84.4 (SD 13.6)	94.9 (SD 12.8)	<0.001
Hip (n = 1869)	102.7 (SD 10.7)	102.9 (SD 11.5)	102.5 (SD 8.6)	NS
WHR (n = 1869)	0.87 (SD 0.09)	0.82 (SD 0.08)	0.92 (SD 0.07)	<0.001
WHtR (n = 1844)	0.53 (SD 0.08)	0.52 (SD 0.09)	0.54 (SD 0.08)	<0.001
%BF (n = 1901)	31.5 (SD 8.5)	35.8 (SD 7.8)	26.9 (SD 6.6)	<0.001
Lung Age (n = 1856)	50.8 (SD 20.5)	47.6 (SD 16.7)	54.1 (SD 23.3)	<0.001
Physical Activity (n = 1901)				
Light	128 (6.7%)	61 (6.3%)	67 (7.2%)	NS
Moderate	481 (25.3%)	231 (23.7%)	250 (27%)	
Intensity	1292 (68%)	683 (70.1%)	609 (65.8%)	
Smoking habit (n =1899)				
Non-Smoker	929 (48.9%)	458 (47%)	471 (50.9%)	<0.001
Ex-smoker	318 (16.7%)	126 (12.9%)	192 (20.8%)	
Smoker	652 (34.3%)	390 (40%)	262 (28.3%)	
Glucose (n = 1886)	99.5 (SD 27.9)	98.6 (SD 28.5)	100.6 (SD 27.2)	NS
HDL (n = 1878)	63.2 (SD 14.5)	66.5 (SD 14.6)	59.7 (SD 13.4)	<0.001
Triglycerides (n = 1881)	108.3 (SD 68.3)	96.6 (SD 54.3)	120.6 (SD 78.7)	<0.001
HBP (n = 1900)	740 (38.9%)	263 (27%)	477 (51.5%)	<0.001
Type II DM (n = 1886)	114 (6%)	44 (4.5%)	70 (7.7%)	<0.01
Metabolic Syndrome (n = 1860)	340 (18.3%)	153 (16%)	187 (20.6%)	<0.05
LD (n = 1856)	472 (25.4%)	216 (23%)	256 (27.9%)	<0.05
Restrictive	250 (13.5%)	122 (13%)	128 (14%)	<0.01
Obstructive	165 (8.9%)	76 (8.1%)	89 (9.7%)	
Mixed	57 (3.1%)	18 (1.9%)	39 (4.3%)	

BMI: Body Mass Index; WHR: Waist to hip ratio; WHtR: Waist to height ratio; BF: Body fat; DM: Diabetes Mellitus; HBP: High Blood Pressure; LD: Lung Dysfunction; NS: Non-significative.

### Metabolic syndrome and lung dysfunction

The mean chronological age was significantly lower in workers suffering from metabolic syndrome (MetS[yes]) than in those who did not (MetS[no]) (p < 0.001). However, the mean lung age was 62.7 ± 19.1 years in the MetS[yes] compared to 48.3 ± 19.1 years in the MetS[no] (p < 0.001).

Introducing in the analysis of covariance (ANCOVA) the chronological age, the differences between both groups remained statistically significant (MetS[yes] vs MetS[no]: 59.4 ± 18.7 years vs 49 ± 18.4 years; p < 0.001) (Table 2). Also, analyzing the difference between lung age and chronological age, it was found that it was significantly higher in MetS[yes] (Table 2).

**Table 2:** Metabolic Syndrome and Lung Age.

Variable	MetS [Yes] (n = 329)		MetS [No] (n = 1487)		p
	Mean (SD)	95% CI	Mean (SD)	95%CI	
Chronological Age	42.6 (10.8)	41.4–43.8	48.7 (9.5)	48.2–49.2	<0.001
Lung Age	62.7 (19.1)	60.6–64.8	48.3 (19.1)	47.3–49.3	<0.001
L. Age – C. Age	14.1 (20.3)	11.9–16.3	5.6 (18.3)	4.7–6.5	<0.001
	**Metabolic Syndrome and Lung Age (ANCOVA)**				
	**Mean (SD)**	**95% CI**	**Mean (SD)**	**95%CI**	
Lung Age	59.4 (18.7)	57.4–61.5	49 (18.4)	48.1–49.9	<0.001*
L. Age – C. Age	15.6 (18.7)	13.6–17.6	5.2 (18.4)	4.3–6.2	<0.001**

ANCOVA model adjusted by chronological age; *R^2^ adjusted = 0.194; Size effect MetS: 0.044; **R^2^ adjusted = 0.062; Size effect MetS: 0.044; L. Age – C. Age: Difference between Lung Age and Chronological Age; MetS: Metabolic Syndrome.

The study of lung dysfunction showed that these were more prevalent among workers suffering from MetS (p < 0.01) (Table 3). Focus the analysis on MetS[yes], the restrictive pattern was observed in a higher proportion (23.4%) than the obstructive (10.3%) and the mixed patterns (7.3%) (p < 0.001).

**Table 3:** Relation between Metabolic Syndrome and Lung Dysfunction.

Variables	Total (n = 1816)	MetS [Yes] (n = 329)	MetS [No] (n = 1487)	p
Lung Dysfunction	461 (25.4%)	135 (41%)	326 (21.9%)	<0.001
Restrictive	250 (13.5%)	77 (23.4%)	166 (11,2%)	<0.001
Obstructive	165 (8.9%)	34 (10.3%)	127 (8.6%)	NS
Mixed	57 (3.1%)	24 (7.3%)	33 (2.2%)	<0.001

NS: Non-significative.

### Lung age and independent variables

The multiple linear regressions showed that the WHR (β = 42.411, t (1817) = 8.874; p < 0.001) and the WHtR (β = 41.438, t (1817) = 7.594; p < 0.001) were the variables that were best associated with lung age. With respect to pathologies, the results evidenced that MetS (β = 9.919, t (1817) = 8.727; p < 0.001) was the one that was most associated with lung aging, followed by type 2 Diabetes Mellitus (β = 9.041, t (1817) = 4.942; p < 0.001) and obesity (BMI ≥ 30 Kg/m^2^) (β = 5.993, t (1817) = 5.870; p < 0.001).

The variation estimated for lung age showed, to equality of the rest of variables, an increase of 4.2 and 4.1 years for each increase in 0.1 of the WHR and WHtR, respectively. Also, it was observed that a WHtR equal to or higher than 0.55 was associated with a lung aging of 64 years (Table 4).

**Table 4:** Lineal multiple regression for Lung Age.

Variable	β	Standardized β	t	R^2^ adjusted	p
Body Mass Index	0.374	0.099	4.697	0.190	<0.001
Waist	0.271	0.187	8.605	0.205	<0.001
Hip	0.157	0.078	3.677	0.187	<0.001
Waist to Hip Ratio	42.411	0.201	8.874	0.207	<0.001
Waist to Height Ratio	41.438	0.165	7.594	0.206	<0.001
Waist to Height Ratio ≥ 0.55	6.393	0.152	7.056	0.202	<0.001
% Body Fat	0.312	0.129	4.697	0.190	<0.001
Glucose	0.101	0.139	6.551	0.197	<0.001
HDL	−0.163	−0.115	−5.222	0.189	<0.001
Triglycerides	0.049	0.164	7.569	0.203	<0.001
Obesity	5.993	0.123	5.870	0.195	<0.001
High Blood Pressure	2.767	0.066	2.854	0.184	<0.001
Diabetes Mellitus type 2	9.041	0.105	4.942	0.189	<0.001
Metabolic Syndrome	9.919	0.187	8.727	0.212	<0.001

Models adjusted by age, smoking habit, sex and physical activity (dichotomized: light and active (moderate and intensity).

In extending the analysis to the clinical categories of the WHtR, it was found that workers who had a WHtR ≥ 0.55 presented significantly greater lung aging (p < 0.001) (Table 5).

**Table 5:** WHtR Clinical Categories and lung age.

Variable	≤0.5	0.51–0.54	≥0.55	p
Lung Age	44.6 (17.3)	50.1 (20)	57.2 (21.6)	<0.001
L. Age – C. Age	5.2 (18.1)*	5.1 (18.7)*	10.1 (19.4)	<0.01

*Without significant differences in post-hoc tests; L. Age – C. Age: Difference between Lung Age and Chronological Age; WHtR: Weight to height ratio.

The prevalence of global lung dysfunction, restrictive and mixed patterns was significantly higher in group WHtR ≥ 0.55 (31.8%, 18.9%, 4.8%), than in 0.51–0.54 (23.2%, 9.9%, 3.4%) and ≤0.5 (20%, 10%, 1.2%) (p < 0.001). Also, Table 6 exhibits a significant linear trend in the prevalence ratio of lung dysfunction and its types and the clinical categories of WHtR (p < 0.001), except for in the obstructive alteration.

**Table 6:** Prevalence ratios for WHtR clinical categories.

Variable	≤0.5	0.51–0.54	≥0.55	p	p
Lung Dysfunction	1 (Ref.)	1.2 (0.9–1.5)	1.6 (1.3–1.9)	<0.001	<0.001
Restrictive	1 (Ref.)	1 (0.7–1.4)	1.9 (1.5–2.5)	<0.001	<0.001
Obstructive	1 (Ref.)	1.1 (0.8–1.6)	0.9 (0.7–1.3)	NS	NS
Mixed	1 (Ref.)	2.9 (1.3–6.9)	4.1 (1.9–8.8)	<0.001	<0.001

Ref.: Reference; L. Age – C. Age: Difference between Lung Age and Chronological Age; WHtR: Weight to height ratio; NS: Non-significative.

## Discussion

The study aimed to determine whether the MetS is associated with respiratory dysfunction as measured by lung age; to evaluate what pulmonary dysfunction is more prevalent among those who suffer from MetS; and what MetS components, variables and pathologies are significantly associated with lung aging in the adult population.

Lung aging is a natural event that can be aggravated by local or systemic pathological processes, being the lung age a sensitive variable to them^18^. About systemic processes, morbid obesity (BMI ≥ 40 kg/m^2^) has been linked to accelerated lung aging^19^. In this respect, our results showed that elevation of BMI (β = 0.374; p < 0.001) and obesity (BMI ≥ 30 kg/m^2^; β = 5.993; p < 0.001) were associated with increased lung age. These results agree with those reported by Mehari *et al*., who found that an increase in BMI produced a reduction in the lung volume in the Afro-American population^28^.

The association between the alteration of the lung function and the MetS has been widely demonstrated^6,7,29^. In this sense, Cheng *et al*. showed that the presence of MetS caused a significant reduction in FVC% of predicted and FEV_1_% of predicted^30^. Yong *et al*. observed that, in the lowest quartile of both parameters, there was an increase in MetS cases^31^. According to these results, our study showed that workers with MetS had a higher mean lung age (59.4 ± 18.7 vs 49 ± 18.4 years; p < 0.001) and a greater difference between this and their chronological age (15.6 ± 18.7 vs. 5.2 ± 18.4 years; p < 0.001). In addition, it is important to note that, according to our results, suffering from MetS was associated to a greater extent with lung aging (β = 9.919; p < 0.001) than with obesity (BMI ≥ 30 Kg/m^2^; β = 5.993; p < 0.001). These results are consistent with those found in other studies, where a higher impact on pulmonary function when metabolic alterations are present with or without obesity, have been evidenced^32,33^.

These alterations in spirometric values due to MetS are clinically relevant through the appearance of pulmonary diseases^6^. In our study population, the total prevalence of lung dysfunction rose to 25.4%, with a significantly higher proportion among individuals suffering from MetS (41% vs 21.9%; p < 0.001). Furthermore, it is worth noting that the restrictive impairment was the most observed in this group (23.4%), a fact that the literature has amply evidenced^6,34^. Related to this, Ford *et al*. found that the prevalence rate of restrictive pattern was higher among those with MetS not occurring with the obstructive^34^.

Moreover, the components of the MetS have been related to the alteration of the spirometric values^30^. This fact is consistent with our findings since the change in the normal values of each component was associated, at a different magnitude, with an accelerated lung aging.

However, abdominal adiposity has been presented as a marker that permits the early detection of alteration in pulmonary function in different populations, emphasizing WC. Our findings have evidenced that the increase of the WC causes an increment in the lung age (β = 0.374; p < 0.001), mediated by the affectation of the FVC implicated in its calculation. These results are consistent with those shown by other authors, pointing that the WC is that which has shown the strongest correlation with the reduction of spirometric values, and it is the most suitable variable for lung function prediction in general and, in particular, for the restrictive impairment^20,33,35–37^.

Nevertheless, the authors recommend the use of the WHtR as it permits the evaluation of the variation in WC in relation to height, and it is associated with the appearance of cardiovascular diseases^21^. In addition, the literature has evidenced its predictive capacity in the diagnosis of MetS, with its highest validity index at the cut-off point of 0.55^12,13^. With respect to lung dysfunction, our results showed that the increase in WHtR was related to accelerated lung aging (β = 42.411; p < 0.001).

The WHtR ≥0.55 increased lung age by 6.4 years and presented a difference between lung age and chronological age of 10 years compared to 5 years for the WHtR <0.55. It should be pointed out that although the subjects with MetS showed aging of 9 years concerning those without it, only the WHtR was responsible for 6 of those 9 years. Namely, the abdominal adiposity (measured by the WHtR) was responsible for 66.7% of lung aging caused by MetS.

Although no studies have evaluated this cut-off point so far, the literature has shown that WHtR is related to the appearance of pulmonary function impairment and that shows the strongest correlation with FEV_1_ and FCV in different populations^21,22,33,38,39^. Also, on studying WHtR according to clinical categories, we observed a significant linear trend in prevalence ratios for global lung dysfunction and restrictive and mixed patterns.

This trend was not observed for obstructive lung impairment. This fact could be explained by the relationship between insulin resistance and abdominal obesity, which can be measured by WHtR. Insulin resistance is one of the pathophysiological mechanisms associated with restrictive alteration and MetS. However, this resistance does not appear in the obstructive alteration^34^.

Early detection of pulmonary disorders and MetS is fundamental to prevent the appearance of pathologies secondary to them^8,40–42^. For this reason, the WHtR is recommended as a marker of central obesity, the main axis in the development of the pathologies mentioned above^12,13,29^. The fact that it is calculated non-invasively, its easy measurement and it has a cut-off point (WHtR ≥ 0.55) that permits to predict the patients’ cardiovascular and lung health status, turns it into a cost-effective tool that can allow early approaches in the clinical setting.

Finally, lung age has been shown to be sensitive to changes in lung function mediated by MetS and its components. In our opinion, this finding allows it to be considered as a valid clinical indicator that makes it possible for patients to know the improvement of their lung health visually^18,19^.

### Limitations

There is no study linking lung age to MetS, which made it difficult to compare ours results with other populations. However, we believe that this line of research could give interesting results in clinical settings, as well as facilitate the interpretation of results by healthcare professionals and patients.

## Conclusions

The MetS caused accelerated lung aging, and a close relationship was found between the later and the appearance of a restrictive lung impairment pattern. The lung age and its difference with the chronological age, have been evidenced as reference variables that permit to find out the respiratory health status simply and intuitively.

The WHtR proved to be the best variable to predict lung dysfunction since the rise in its value was associated with a higher lung age and an increase in the prevalence of pulmonary impairment. In particular, individuals with a WHtR ≥ 0.55 presented significantly worse lung health than those with WHtR < 0.55.

## References

[1] Alberti K (2009). Harmonizing the metabolic syndrome. Circulation..

[2] Fernández-Bergés D (2012). Síndrome metabólico en España: prevalencia y riesgo coronario asociado a la definición armonizada y a la propuesta por la OMS. Estudio DARIOS. Rev Esp Cardiol..

[3] Shin D, Kongpakpaisarn K, Bohra C (2018). Trends in the prevalence of metabolic syndrome and its components in the United States 2007–2014. Int J Cardiol..

[4] Jonk A (2007). Microvascular Dysfunction in Obesity: A Potential Mechanism in the Pathogenesis of Obesity-Associated Insulin Resistance and Hypertension. Physiology (Bethesda)..

[5] Mendrick D (2018). Metabolic Syndrome and Associated Diseases: From the Bench to the Clinic. Toxicol Sci..

[6] Baffi C (2016). Metabolic Syndrome and the Lung. Chest..

[7] Lee, Y. *et al*. Association between HOMA-IR and Lung Function in Korean Young Adults based on the Korea National Health and Nutrition Examination Survey. *Scientific Reports*. **7**(1) (2017). 10.1038/s41598-017-11337-3 PMC5601425 28916790

[8] Padberg I (2018). Pulmonary dysfunction and development of different cardiovascular outcomes in the general population. Arch Cardiovasc Dis..

[9] World Health Organization. The top 10 causes of death. 2019 Aviable at, https://www.who.int/news-room/fact-sheets/detail/the-top-10-causes-of-death [Accessed 6 January 2020].

[10] Global Burden of Cardiovascular Diseases Among US States, 1990–2016 (2018). The Burden of Cardiovascular Diseases Among US States, 1990-2016. JAMA Cardiol..

[11] Wilkins, E. *et al*. European Cardiovascular Disease Statistics 2017. European Heart Network, Brussels.

[12] Romero-Saldaña M (2016). New non-invasive method for early detection of metabolic syndrome in the working population. Eur J Cardiovasc Nurs..

[13] Romero-Saldaña M (2018). Validation of a non-invasive method for the early detection of metabolic syndrome: a diagnostic accuracy test in a working population. BMJ Open..

[14] Martin C (2017). Validación de las ecuaciones de referencia para la espirometría forzada en niños sanos preescolares españoles. Arch Bronconeumol..

[15] Quanjer P (2012). Multi-Ethnic Reference Values For Spirometry For The 3–95 Year Age Range: The Global Lung Function 2012 Equations: Report of the Global Lung Function Initiative (GLI), ERS Task Force to establish improved Lung Function Reference Values. Eur Respir J..

[16] Quanjer PH, Brazzale DJ, Boros PW, Pretto JJ (2013). Implications of adopting the Global Lungs Initiative 2012 all-age reference equations for spirometry. Eur Respir J..

[17] Morris J, Temple W (1985). Spirometric “Lung Age” Estimation for Motivating Smoking Cessation. Preventive Medicine..

[18] Castrejón-Vázquez M (2014). Pulmonary age-chronological age relation as indicator of improvement and severity of patients with bronchial asthma. Rev Alerg Mex..

[19] D’Ávila SM (2010). Accelerated lung aging in patients with morbid obesity. J Bras Pneumol..

[20] Wehrmeister FC (2012). Waist circumference and pulmonary function: a systematic review and meta-analysis. Syst Rev..

[21] Solí-Aguilar M (2016). Adiposity markers and lung function in smokers: a cross-sectional study in a Mediterranean population. BMC Pulm Med..

[22] Kjer K (2018). The associations between weight-related anthropometrics during childhood and lung function in late childhood: a retrospective cohort study. BMC Pulm Med..

[23] García-Rio, F. & Calle, M. Normativa sobre la espirometría (revisión 2013). Barcelona: Respira (2013).

[24] Ashwell M, Hsieh SD (2005). Six reasons why the waist-to-height ratio is a rapid and effective global indicator for health risks of obesity and how its use could simplify the international public health message on obesity. Int J Food Sci Nutr..

[25] Callaway, C.W. *et al*. Circumferences. In: Lohman, T, G., Roche, A. F. & Martorell, R. (eds) *Anthropometric Standardization Reference Manual*. Campaign: Human Kinetics Books, pp. 44–45 (1991).

[26] González, A. La medición correcta de la presión arterial. In: *Manual de hipertensión arterial en la práctica clínica de atención primaria*. Grupo de hipertensión arterial. Sociedad Andaluza de Medicina Familiar y Comunitaria, pp. 35–41 (2006).

[27] Molina-Luque, R. *et al*. Equation Córdoba: A Simplified Method for Estimation of Body Fat (ECORE-BF). *Int J Environ Res Public Health*. **16**(22), pii: E4529; 10.3390/ijerph16224529 (2019). 10.3390/ijerph16224529 PMC6888348 31731813

[28] Mehari A (2015). Obesity and Pulmonary Function in African Americans. PLoS One..

[29] Sagun G (2015). The relation between insulin resistance and lung function: a cross sectional study. BMC Pulm Med..

[30] Chen WL (2014). Relationship between lung function and metabolic syndrome. PLoS One..

[31] Yong S, Rhree EJ, Sung KC (2010). Metabolic Syndrome, Insulin Resistance and Systemic Inflammation as Risk Factors for Reduced Lung Function in Korean Nonsmoking Males. J Korean Med Sci..

[32] Lee H (2019). Metabolic health is more closely associated with decrease in lung function than obesity. PLoS One..

[33] Cardet J, Ash S, Kusa T, Camargo C, Israel E (2016). Insulin resistance modifies the association between obesity and current asthma in adults. Eur Respir J..

[34] Ford E, Cunningham T, Mercado C (2014). Lung function and metabolic syndrome: Findings of National Health and Nutrition Examination Survey 2007–2010. J Diabetes..

[35] Caspersen N (2018). The association between circulating adiponectin levels, lung function and adiposity in subjects from the general population; data from the Akershus Sleep Apnea Project. BMC Pulm Med..

[36] Vatrella A (2016). Abdominal adiposity is an early marker of pulmonary function impairment: Findings from a Mediterranean Italian female cohort. Nutr Metab Cardiovasc Dis..

[37] Rowe A, Hernandez P, Kuhle S, Kirkland S (2017). The association between anthropometric measures and lung function in a population-based study of Canadian adults. Respir Med..

[38] Sadeghimakki R, McCarthy D (2019). Interactive effects of adiposity and insulin resistance on the impaired lung function in asthmatic adults: cross-sectional analysis of NHANES data. Ann Hum Biol..

[39] Choudhuri D, Sutradhar B (2015). Pulmonary function of adolescents from Tripura, a North-eastern state of India. Lung India..

[40] Lee K (2017). Lung function and impaired kidney function in relation to metabolic syndrome. Int Urol Nephrol..

[41] Moualla M (2017). Rapid decline in lung function is temporally associated with greater metabolically active adiposity in a longitudinal study of healthy adults. Thorax..

[42] Yamamoto Y, Oya J, Tomoko N, Uchigata Y (2014). Association between lung function and metabolic syndrome independent of insulin in Japanese men and women. Jpn Clin Med..

